# Effect of a Mobile App for the Pharmacotherapeutic Follow-Up of Patients With Cancer on Their Health Outcomes: Quasi-Experimental Study

**DOI:** 10.2196/20480

**Published:** 2020-10-16

**Authors:** Roberto Collado-Borrell, Vicente Escudero-Vilaplana, Almudena Ribed, Cristina Gonzalez-Anleo, Maite Martin-Conde, Rosa Romero-Jimenez, Irene Iglesias-Peinado, Ana Herranz-Alonso, Maria Sanjurjo-Saez

**Affiliations:** 1 Hospital General Universitario Gregorio Marañon Madrid Spain; 2 Hospital Clinic Barcelona Barcelona Spain; 3 Faculty of Pharmacy, Universidad Complutense de Madrid Madrid Spain

**Keywords:** e-OncoSalud, app, smartphone, oral antineoplastic agent, oncology

## Abstract

**Background:**

Oral antineoplastic agents (OAAs) have revolutionized cancer management. However, they have been reported with adverse side effects and drug-drug interactions. Moreover, patient adherence to OAA treatment is critical. Mobile apps can enable remote and real-time pharmacotherapeutic monitoring of patients, while also promoting patient autonomy in their health care.

**Objective:**

The primary objective was to analyze the effect of using a mobile app for the follow-up of patients with oncohematological malignancies undergoing treatment with OAAs on their health outcomes. The secondary objectives were to analyze the role of the app in communication with health care professionals and patient satisfaction with the app.

**Methods:**

We performed a comparative, quasi-experimental study based on a prepost intervention with 101 patients (control group, n=51, traditional pharmacotherapeutic follow-up vs intervention group, n=50, follow-up through e-OncoSalud, a custom-designed app that promotes follow-up at home and the safety of patients receiving OAAs). The effect of this app on drug safety, adherence to treatment, and quality of life was evaluated.

**Results:**

With regard to drug safety, 73% (37/51) of the patients in the control group and 70% (35/50) of the patients in the intervention group (*P*=.01) presented with drug-related problems. The probability of detecting an insufficiently treated health problem in the intervention group was significantly higher than that in the control group (*P*=.04). The proportion of patients who presented with side effects in the intervention group was significantly lower than that in the control group (*P*>.99). In the control group, 49% (25/51) of the patients consumed some health resources during the first 6 months of treatment compared with 36% (18/50) of the patients in the intervention group (*P*=.76). Adherence to treatment was 97.6% (SD 7.9) in the intervention group, which was significantly higher than that in the control group (92.9% [SD 10.0]; *P*=.02). The EuroQol-5D in the intervention group yielded a mean (SD) index of 0.875 (0.156), which was significantly higher than that in the control group (0.741 [0.177]; *P*<.001). Approximately 60% (29/50) of the patients used the messaging module to communicate with pharmacists. The most frequent types of messages were acknowledgments (77/283, 27.2%), doubts about contraindications and interactions with OAAs (70/283, 24.7%), and consultations for adverse reactions to treatment (39/283, 13.8%). The satisfaction with the app survey conducted in the intervention group yielded an overall mean (SD) score of 9.1 (0.4) out of 10.

**Conclusions:**

Use of e-OncoSalud for the real-time follow-up of patients receiving OAAs facilitated the optimization of some health outcomes. The intervention group had significantly higher health-related quality of life and adherence to treatment than the control group. Further, the probability of the intervention group presenting with side effects was significantly lower than that of the control group.

## Introduction

Cancer research has grown exponentially in recent years. It is currently estimated that 40% of the chemotherapeutic drugs are oral antineoplastic agents (OAAs), which have changed the model for administration of chemotherapy. The treatment for cancer has changed from controlled administration in the day hospital to administration at home without the supervision of a health care professional, thus requiring greater autonomy on the part of the patient [1,2]. However, these treatments are subject to drug-related problems (DRPs), such as adherence, interactions with the usual medication, and side effects [3,4]. The Institute for Safe Medication Practices classifies OAAs as high-risk medications [5]. According to Walsh et al [6], up to 20% of the patients treated with OAAs experience severe side effects.

In this sense, information and communication technologies, specifically the area of mobile health, can provide patients with greater autonomy and facilitate communication with health care professionals. Similarly, mobile health can enable health care professionals to improve monitoring and patient care [7]. According to a study in more than 7800 patients, 83% considered the use of technology to be an essential or very important component of their health care. The majority valued the use of virtual care very positively in various scenarios such as receiving reminders for health promotion, taking medication, changing an appointment, and even follow-up on discharge and contacting their health care professional [8].

Since mobile apps are accessible by a vast majority of the population and since these apps facilitate the possibility of remote monitoring, they offer patients with cancer the opportunity to participate in the management of their disease and endow greater responsibility for the control of their health, thus favoring empowerment and improving the safety and quality of care [7].

According to the report “Global Oncology Trends 2018,” it is estimated that there are more than 2500 mobile health cancer-related apps and that their use in clinical practice is increasing, especially in the case of health care apps [9]. Nevertheless, evidence for the benefits that these apps can bring to patients is limited. In fact, 2015 was the first year that this advancement was addressed in the literature, with 5 studies analyzing the effectiveness of apps in oncology. All 5 studies reported positive results, although 2 showed efficacy similar to that in the standard of care. In a review of 17 studies on the use of apps and websites for patients with cancer, it was observed that most were focused exclusively on the design, feasibility, and acceptance of the app. There is hardly any evidence on the effect of apps intended for the patient on the improvement of their health outcomes [10].

Therefore, the objective of this study was to analyze the effect of an app for the pharmacotherapeutic follow-up of patients with oncohematological malignancies undergoing treatment with OAAs on their health outcomes. The secondary objective was to analyze the role of the app in communication with health care professionals and patient satisfaction with the app.

## Methods

### Design and Scope of the Study

We performed a comparative, quasi-experimental study with a prepost intervention design. The health outcomes of a group of patients in which pharmacotherapeutic follow-up was according to the usual clinical practice of the Pharmacy Service (control group) was compared with those of a group of patients in which the said follow-up was managed through an app (intervention group).

There were 2 periods of recruitment: the first began in January 2015 and ended in December 2015 (control group) and the second began in May 2017 and ended in May 2018 (intervention group). The follow-up period in both the groups was 6 months. This study was carried out in the outpatient unit of the Pharmacy Services of 2 University Hospitals. Both services have a pharmaceutical care program for patients undergoing treatment with OAAs to train them in the appropriate management of their medication (optimal adherence, management of side effects, and interactions with usual medication). This study was approved by the Hospital Clinical Research Ethics Committee (code PI13/02056). All patients signed an informed consent document. This study adhered to the basic ethics principles and the tenets of the Declaration of Helsinki.

### Study Population

The control group consisted of adult patients who started treatment with OAAs between January 1, 2015 and December 31, 2015. These patients were followed up at the outpatient unit at the beginning of the treatment and 6 months later according to the established pharmaceutical care program. The intervention group consisted of patients over 18 years of age who started treatment with OAAs between May 31, 2017 and May 31, 2018. Patients had to have a smartphone and were followed up daily through an app (e-OncoSalud) for 6 months from the start of the treatment.

### Pharmacotherapeutic Follow-Up Via the App

Between January 2016 and May 2017, a team of hospital pharmacists, oncologists, hematologists, and computer scientists designed the e-OncoSalud app [2]. e-OncoSalud is a custom-designed app that promotes pharmacotherapeutic follow-up at home and the safety of patients treated with OAAs. It comprises the following 5 modules that integrate all relevant treatment information.

1. An agenda module, wherein the patient can register various events (appointment with the doctor, laboratory analyses, imaging test appointments, and medication collection) with customizable alerts.

2. A treatment module, in which both the patient and the pharmacist can register the drugs consumed by the patient and the dosage. Patients can see their package insert and schedule alerts for administration, thus improving adherence.

3. A module providing tips on the disease and management of symptoms, as well as links to websites of interest and instructions for using the app.

4. A messaging module so that both the patient and the health care professional can contact each other at any time.

5. A module in which the patient can record general progress, blood pressure, weight, and side effects. The patient can register these with a defined periodicity, except for the side effects, which are recorded when they occur. The management of the side effects is based on a decision algorithm that uses a series of questions to classify severity according to the severity of the Common Terminology Criteria for Adverse Events (CTCAE, grades 1-4) and issues appropriate recommendations. The side effect information is focused on the management of fatigue, diarrhea, nausea, vomiting, hand-foot syndrome, and fever. In diarrhea and vomiting, the decision algorithm works based on a count of the number of events registered in the last 24 hours, and when it reaches a defined number of events, the different recommendations appear. All the information that the patient records in the app is sent through a web interface to ensure real-time pharmacotherapeutic follow-up by pharmacists.

### Variables

The effect of the app was evaluated through the following health outcomes: drug safety, adherence, and quality of life. Patient communication and satisfaction with the app were also analyzed. The variables analyzed were as follows.

1. Demographic/clinical characteristics: age, sex, diagnosis, type of OAA, and concomitant medications.

2. Safety: DRPs according to the 3rd Consensus of Granada, the number of interactions and their severity, side effects according to the CTCAE scale v.4.03 [11], and resource consumption (unscheduled consultations, emergency visits, and hospital admissions).

3. Quality of life: assessed using the EuroQol-5D (EQ-5D) questionnaire [12].

4. Adherence to treatment: measured through the medication possession ratio, calculated through the dispensation record of the outpatient unit’s computer program.

5. Communication: analysis of the messages that patients sent through the app.

6. Satisfaction: evaluated through a specific satisfaction survey for the app. The survey consisted of 8 closed questions on an additive assessment scale that included aspects of the ease of communication with the pharmacist, usefulness for managing the treatment, and grade of recommendation. The survey was delivered to patients 6 months after having installed the app.

### Sample Size

The objective of the app was to improve the pharmaceutical follow-up and safety of patients treated with OAAs by increasing the detection of DRPs by at least 20% (from 15% to 35%). Therefore, accepting an alpha risk of .05 and a beta risk of .10 in a bilateral contrast, 45 subjects are needed in the control group and 45 in the intervention group. A loss to follow-up rate of 10% has been estimated.

### Statistical Analysis

Data were analyzed using SPSS Statistics for Windows, version 21.0 (IBM Corp). The results were expressed as mean and standard deviation (SD). Categorical variables were expressed as frequencies and percentages. The homogeneity of the 2 groups was analyzed using a univariate analysis by applying the chi-square test for qualitative variables and the one-sided *t* test or Mann-Whitney *U* test to compare quantitative variables. The Bonferroni correction was applied for multiple comparisons. *P* values less than .05 were considered statistically significant.

## Results

### Demographic Data of the Patients

**Figure 1:**
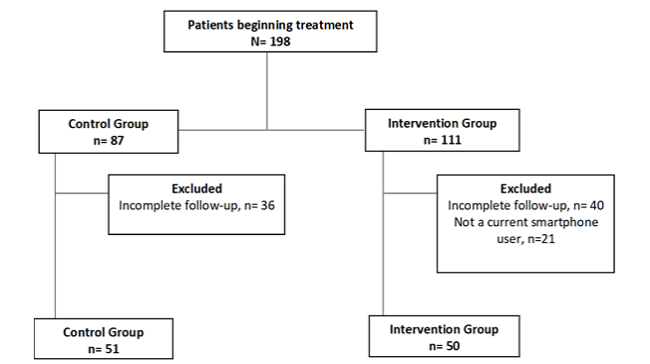
Description of the recruitment of the patients.

During the study period, 198 patients started treatment with OAAs (Figure 1). A total of 87 patients were assigned to the control group; of these, 36 were excluded because they did not complete the 6 months of treatment. In the intervention group, 111 patients started treatment but 61 patients were excluded (40 for not completing the follow-up period and 21 for not having a smartphone). Finally, 101 patients were analyzed in a 1:1 ratio, with 51 patients in the control group (without the app) and 50 patients in the intervention group (with the app). The mean (SD) age of the patients was 62.7 (13.6) years: 68.7 (10.7) years in the control group and 56.6 (13.6) years in the intervention group (*P*<.001). Table 1 describes the demographic and clinical characteristics of the study patients.

**Table 1:** Demographic and clinical characteristics of the control group and the intervention group.

Demographic/clinical characteristics		Control group, n=51, n (%)	Intervention group, n=50, n (%)	Total, N=101, n (%)		*P* value
**Gender**					.20	
	Female	19 (37)	25 (50)	44 (43.6)		
	Male	32 (63)	25 (50)	57 (56.4)		
**Tumor**					.004^a^	
	Multiple myeloma	13 (26)	5 (10)^a^	18 (17.8)		
	Non–small cell lung cancer	12 (24)	5 (10)	17 (16.8)		
	Kidney cancer	11 (22)	4 (8)	15 (14.9)		
	Prostate cancer	5 (10)	6 (12)	11 (10.9)		
	Chronic myeloid leukemia	2 (4)	4 (8)	6 (5.9)		
	Breast cancer	3 (6)	2 (4)	5 (5.0)		
	Gastrointestinal stromal tumors	1 (2)	3 (6)	4 (4.0)		
	Soft tissue sarcoma	0 (0)	4 (8)	4 (4.0)		
	Hepatocellular carcinoma	0 (0)	3 (6)	3 (3.0)		
	Central nervous system	0 (0)	3 (6)	3 (3.0)		
	Thyroid cancer	0 (0)	3 (6)	3 (3.0)		
	Chronic lymphatic leukemia	0 (0)	3 (6)	3 (3.0)		
	Colon cancer	2 (4)	0 (0)	2 (2.0)		
	Myelodysplastic syndrome	2 (4)	0 (0)	2 (2.0)		
	Ovarian cancer	0 (0)	2 (4)	2 (2.0)		
	Acute lymphoid leukemia	0 (0)	1 (2)	1 (1.0)		
	Melanoma	0 (0)	1 (2)	1 (1.0)		
	Other	0 (0)	1 (2)	1 (1.0)		
**Eastern Cooperative Oncology Group performance status**					.04	
	Grade 0	20 (41)	31 (66)	51 (53.1)		
	Grade 1	25 (51)	15 (32)	40 (41.7)		
	Grade 2	4 (8)	1 (2)	5 (5.2)		
**Smoker**					.15	
	No	51 (100)	48 (96)	99 (98.0)		
	Yes	0 (0)	2 (4)	2 (2.0)		
**Living alone**					.13	
	No	39 (77)	44 (88)	83 (82.2)		
	Yes	12 (24)	6 (12)	18 (17.8)		
**Treatment**					.001^a^	
	Lenalidomide	15 (29)	4 (8)^a^	19 (18.8)		
	Pazopanib	8 (16)	3 (6)	11 (10.9)		
	Gefitinib	7 (14)	1 (2)^a^	8 (7.9)		
	Imatinib	4 (8)	4 (8)	8 (7.9)		
	Capecitabine	6 (12)	1 (2)	7 (6.9)		
	Sorafenib	0 (0)	6 (12)	6 (5.9)		
	Erlotinib	5 (10)	0 (0)	5 (5.0)		
	Abiraterone	3 (6)	2 (4)	5 (5.0)		
	Enzalutamide	1 (2)	4 (8)	5 (5.0)		
	Sunitinib	2 (4)	2 (4)	4 (4.0)		
	Dasatinib	0 (0)	3 (6)	3 (3.0)		
	Afatinib	0 (0)	3 (6)	3 (3.0)		
	Ibrutinib	0 (0)	3 (6)	3 (3.0)		
	Olaparib	0 (0)	2 (4)	2 (2.0)		
	Regorafenib	0 (0)	2 (4)	2 (2.0)		
	Procarbazine	0 (0)	2 (4)	2 (2.0)		
	Axitinib	0 (0)	1 (2)	1 (1.0)		
	Crizotinib	0 (0)	1 (2)	1 (1.0)		
	Everolimus	0 (0)	1 (2)	1 (1.0)		
	Ruxolitinib	0 (0)	1 (2)	1 (1.0)		
	Dabrafenib/trametinib	0 (0)	1 (2)	1 (1.0)		
	Temozolomide	0 (0)	1 (2)	1 (1.0)		
	Lenvatinib	0 (0)	1 (2)	1 (1.0)		
**Treatment line^b^**					.61	
	1	26 (53)	27 (54)	53 (53.5)		
	2	14 (29)	14 (28)	28 (28.3)		
	3	8 (16)	5 (10)	13 (13.1)		
	4	1 (2)	3 (6)	4 (4.0)		
	5	0 (0)	1 (2)	1 (1.0)		
**Previous oral chemotherapy**					.15	
	No	36 (72)	42 (84)	78 (78.0)		
	Yes	14 (28)	8 (16)	22 (22.0)		

^a^Significant difference between the control and the intervention group at *P*<.05.

^b^Number of previous treatments the patient has received.

### Drug Safety

#### DRPs

DRPs were recorded in 73% (37/51) of the patients in the control group and in 70% (35/50) of the patients in the intervention group (*P*=.01) (Table 2). The most frequent DRPs in both groups were interactions with the usual medication. The probability of the intervention group presenting with side effects was significantly lower than that of the control group presenting with side effects (*P*=.01). The probability of detecting an insufficiently treated health problem in the intervention group was significantly higher than that of detecting an insufficiently treated health problem in the control group (*P*=.04).

**Table 2:** Drug-related problems in the control group and intervention group.

Drug-related problem	Control group, n=51, n (%)	Intervention group, n=50, n (%)	Total, N=101, n (%)
Erroneous administration of the drug	2 (3)	3 (5)	5 (3.5)
Personal characteristics	0 (0)	2 (3)	2 (1.4)
Contraindication	0 (0)	2 (3)	2 (1.4)
Dose, schedule, or inadequate duration	3 (4)	2 (3)	5 (3.5)
Prescription errors	0 (0)	1 (2)	1 (0.7)
Nonadherence	9 (11)	6 (9)	15 (10.4)
Drug-drug interactions	26 (33)	21 (32)	47 (32.6)
Other health problems affecting treatment	1 (1)	0 (0)	1 (0.7)
Likelihood of side effects	19 (24)^a^	2 (3)	21 (14.6)
Insufficiently treated health problem	5 (6)	11 (17)^a^	16 (11.1)

^a^Significant difference between the control and the intervention groups at *P*<.05.

#### Side Effects

The proportion of patients who presented with side effects in the group that used the app was significantly lower than that of patients who presented with side effects in the control group (45/50, 90% vs 51/51, 100%; *P*>.99). Table 3 shows the distribution of the patients according to side effects in the control group and intervention group. No statistically significant differences were found between the 2 groups based on side effects. The mean (SD) time to onset of the first adverse effect was 8.2 (10.6) days in the intervention group.

**Table 3:** Distribution of patients according to side effects in the control group and intervention group.

Side effects	Control group, n=51, n (%)	Intervention group, n=50, n (%)	Total, N=101, n (%)
Nausea	12 (24)	26 (28)	38 (37.6)
Vomiting	3 (6)	13 (20)	16 (15.8)
Diarrhea	21 (41)	46 (50)	67 (66.3)
Hypertension	7 (14)	17 (20)	24 (23.8)
Hematologic toxicity	9 (18)	16 (14)	25 (24.8)
Fatigue	27 (53)	56 (58)	83 (82.2)
Hand-foot syndrome	7 (14)	11 (8)	18 (17.8)
Cutaneous adverse effects	16 (31)	34 (36)	50 (49.5)
Others	28 (55)	59 (62)	87 (86.1)

### Resource Consumption

Almost half (25/51, 49%) of the patients in the control group consumed health care resources during the first 6 months of treatment compared with 36% (18/50) of the patients in the intervention group (*P*=.78) (Table 4). Four emergency visits were avoided in the intervention group, since the events were managed remotely. No significant differences were found between the control group and the intervention group with regard to resource consumption.

**Table 4:** Resource consumption in the control and intervention groups.

Resource consumption	Control group, n=51, n (%)	Intervention group, n=50, n (%)	Total, N=101, n (%)
Unscheduled visits to the oncology department	4 (11)	3 (14)	7 (12.3)
Emergency visits	22 (63)	17 (77)	39 (68.4)
Admission to hospital	9 (26)	2 (9)	11 (19.3)

### Health-Related Quality of Life

When a linear transformation was applied to standardize the score, the EQ-5D in the intervention group yielded a mean (SD) index of 0.875 (0.156), which was significantly higher than that in the control group (0.741 [0.177]; *P*<.001).

### Adherence

The mean (SD) rate of adherence to treatment in the intervention group was 97.6% (7.9), which was significantly higher than that in the control group (92.9% [10.0]; *P*=.02)

### Patient Communication Through e-OncoSalud

Approximately 60% (29/50) of the patients in the intervention group used the messaging module to communicate with pharmacists. They sent 283 messages, that is, an average of 8 messages per patient. The most frequent messages concerned doubts about contraindications and interactions with OAAs (70/283, 24.7%), consultations for adverse reactions to treatment (39/283, 13.8%), and acknowledgment of the care received (77/283, 27.2%) (Table 5).

**Table 5:** Classification of the messages sent by patients through e-OncoSalud (n=283).

Message classification	Values, n (%)
Acknowledgment of the pharmaceutical care received	77 (27.2)
Contraindications and drug interactions	70 (24.7)
Side effects	39 (13.8)
Medication collection alert	36 (12.7)
Dosage and administration	24 (8.5)
Hospital logistics	15 (5.3)
Use of therapy and efficacy of other treatments	7 (2.5)
Availability of other treatments	6 (2.1)
App-related problems	3 (1.1)
Suggestions for improving the app	3 (1.1)
Use and efficacy of complementary medicinal products	2 (0.7)
Other	1 (0.4)

### Satisfaction With e-OncoSalud

**Figure 2:**
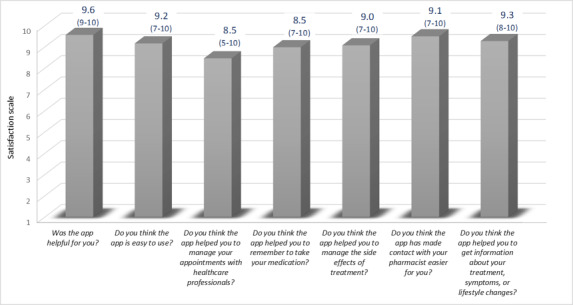
Satisfaction of the patients in the intervention group with the e-OncoSalud app.

The satisfaction survey conducted in the intervention group yielded an overall mean (SD) score of 9.1 (0.4) out of 10. The results are shown in Figure 2.

## Discussion

**Principal Findings and Comparison With Previous Studies**

To our knowledge, this is the first study to show that an app for the pharmacotherapeutic follow-up of patients with cancer treated with OAAs has a positive impact on their safety and adherence to treatment as well as on health-related quality of life. e-OncoSalud is a new app that includes a unique decision-making algorithm. Depending on the type and severity of the side effects registered by the patient, the app provides personalized recommendations instantly.

Although OAAs are not subject to the specific problems of intravenous chemotherapy, they are not without side effects [3,13]. In our study, the most common side effects reported by patients in both groups were characteristic of OAAs (asthenia, diarrhea, skin disorders, nausea, and vomiting) [3]. However, by having the decision-making algorithm, the patients in the intervention group were able to manage these symptoms from home. In a 3-module app [14] aimed at patients with nasopharyngeal carcinoma receiving chemoradiotherapy, one of the recommendations decreased the frequency of the complications associated with treatment. The incidence of mucositis, xerostomia, difficulty opening the mouth, and nasal congestion was lower in the patients who used the app (67 patients) than in those who did not (65 patients) [14]. In a clinical trial conducted in 76 patients with breast cancer [15], the authors analyzed the impact of the ILOVEBREAST app in reducing side effects, improving psychological status, and improving adherence to treatment. The patients were randomized in a 1:1 ratio to follow-up through the app (intervention group) or traditional follow-up (control group). Consistent with our findings, the results showed that the use of the app was associated with better quality of life, greater adherence, and fewer side effects such as nausea, fatigue, hand-foot syndrome, and hair loss. Although the differences were not significant in most cases, the incidence of grade 3 fatigue was significantly lower in patients who used the app (1 vs 13; *P*=.02) [15].

According to a study conducted at the Dana-Farber Cancer Institute, patients undergoing treatment with OAAs are more likely to be admitted to hospital with side effects. However, patients who were closely followed by their health care professional were significantly less likely to be admitted in the hospital [16]. Thus, information and communication technologies are demonstrating an improvement in the management of various conditions, with results that show a reduction in complications and hospital admissions. For example, a study that analyzed the use of text messages in patients with diabetes found a significant decrease in HbA_1c_ levels, improved medication adherence, and decreased emergency department visits [17]. Another study evaluated the effect of home telemonitoring in patients with heart failure and demonstrated a significant improvement in health outcomes [18]. However, we found no studies showing that apps for patients with cancer reduce their emergency visits or hospital admissions. Through e-OncoSalud, patients’ consumption of resources could be reduced from 49% to 36% (unscheduled consultations, emergency visits, or hospital admissions), although there was no statistically significant difference between the control and the intervention groups.

Side effects can also affect the quality of life of patients. According to Rincon et al [19], real-time monitoring and symptom management significantly improved emotional status, insomnia, and urinary tract symptoms. In another study that analyzed an app for the follow-up of patients with lung cancer, registration and follow-up of symptoms and subsequent management by the oncologist improved the quality of life [20]. According to the authors, this improvement was due to the early detection of side effects, complications, and signs or symptoms of progression [20]. In our study, follow-up based on the use of the e-OncoSalud app improved the health-related quality of life more than the traditional follow-up.

While patients with cancer face many challenges, adherence to OAAs is crucial if they are to maximize treatment outcomes and avoid complications [21]. In the follow-up based on e-OncoSalud, adherence at 6 months was 97.6% (SD 7.9), which is significantly higher than that of patients who did not receive home follow-up through the app (92.9% [SD 10.0]). These results agree with those reported in the literature, where the app is positioned as another strategy to improve adherence to treatment [22].

Regarding patient-pharmacist communication, 60% of the patients used the messaging module to communicate with the pharmacist. This percentage is similar to that reported with the WebChoice app [23], in which 61% of the patients used the messaging module to contact their nurse. However, we were unable to find studies that analyze the type of messages sent through an app between patients and health care professionals. This is not surprising, given that few electronic systems for patients with cancer incorporate messaging modules. A recently published review showed that only 6 (15%) of 40 electronic systems incorporated this modality [10]. Although several studies have shown that patients highly value this function, its complexity and maintenance limit its usability [10,24-26].

Finally, our results revealed a high degree of satisfaction with most of the aspects assessed. Those aspects that were the best rated were the ability to communicate with the pharmacist from anywhere, having the treatment registered with the notification system, the immediacy of the response by the pharmacist, and the ease of use. In addition, 100% of the patients agreed that they would recommend the app. These data are similar to those reported in studies that analyzed the app satisfaction of patients with cancer [15,25]. In order to assess the satisfaction of the ILOVEBREAST app, Kim et al [15] conducted a survey consisting of 8 questions that covered the ease of use and help with taking medication. Patients were also asked if they would recommend the app to others. The functions most highly valued by the patients were the ease of obtaining information and managing side effects. However, unlike our app, ease of use was one of the least valued aspects, possibly because it is an app based on an avatar type game. In their evaluation of the iCancerHealth app, Berry et al [25] observed that the aspects best valued by patients were ease of communication with their health care professional and the recording and monitoring of side effects.

### Limitations

This study has the following limitations. The main limitation is the absence of randomization. However, the inclusion and exclusion criteria ensured that the selection of patients was representative of a usual clinical practice, and the sample size was sufficiently large to achieve the objectives. Likewise, the control and intervention groups were recruited 3 years apart. This could have an impact on not only which antineoplastic drugs are available but also other factors in terms of people’s willingness to communicate via an app. In addition, the use of 2 separate study periods with respect to a type of drugs undergoing development makes it difficult to draw reasonable comparisons between the groups.

### Conclusions

Real-time pharmacotherapeutic follow-up of patients receiving OAAs by using the e-OncoSalud enabled the optimization of some health outcomes. Although there was no significant difference between the control and intervention groups in terms of unscheduled visits to the oncology department, emergency visits, or admissions to the hospital, the intervention group had significantly higher health-related quality of life and adherence to treatment. Further, the probability of the intervention group presenting with side effects was significantly lower than that of the control group. However, more randomized studies should be conducted to confirm the observed findings.

## Abbreviations

CTCAE: Common Terminology Criteria for Adverse Events
DRP: drug-related problem
EQ-5D: EuroQol-5D
OAA: oral antineoplastic agent
